# More alive than dead: non-apoptotic roles for caspases in neuronal development, plasticity and disease

**DOI:** 10.1038/cdd.2017.64

**Published:** 2017-06-23

**Authors:** Amrita Mukherjee, Darren W Williams

**Affiliations:** 1 Centre for Developmental Neurobiology, King’s College London, London, UK

## Abstract

Nervous systems are arguably the most fascinating and complex structures in the known universe. How they are built, changed by experience and then degenerate are some of the biggest questions in biology. Regressive phenomena, such as neuron pruning and programmed cell death, have a key role in the building and maintenance of the nervous systems. Both of these cellular mechanisms deploy the caspase family of protease enzymes. In this review, we highlight the non-apoptotic function of caspases during nervous system development, plasticity and disease, particularly focussing on their role in structural remodelling. We have classified pruning as either macropruning, where complete branches are removed, or micropruning, where individual synapses or dendritic spines are eliminated. Finally we discuss open questions and possible future directions within the field.

## Facts

- Caspases have crucial non-death-related roles in neurons.
- Both macropruning and micropruning events deploy key components of the intrinsic caspase pathway.
- Caspases initiate micropruning events in progressive neurodegenerative diseases as the key proteins involved have emerged as caspase substrates.

## Open Questions

- Are cytochrome *c* or Apaf1 dispensable for non-apoptotic roles?
- Are effector caspases activated *de novo* or stalled and released upon non-apoptotic stimulus?
- What molecular mechanisms restrict the extent and spread of activated caspases?
- From an evolutionary perspective what came first—their use in killing or sculpting?

The nervous system is remarkable not just because of its cellular diversity and elaborate connectivity but also because its structure changes constantly throughout life: growing at first, remodelling from experiences, and then degenerating. Here we focus on the non-apoptotic roles that caspases have in the structural remodelling of neurons.

The size and complexity of neurons gives us an opportunity to explore the fundamental principles of cellular design. Some of the largest cells in the body are the motoneurons that innervate the adductor hallucis muscles in our foot. If the cell body of this class of motoneuron were the size of a Volkswagen Beetle then the axon would be equivalent to a 40 km exhaust pipe. This great distance between the cell body and the distal compartments allows us to tease apart events that would otherwise be difficult to resolve in compact cells such as fibroblasts. The devolved nature of neurons encourages us to think differently about how caspase-based signals propagate.

## The Caspases

Caspases are a highly conserved family of cysteine proteases found in all multicellular animals. Although key regulators of inflammation (reviewed in Jiménez Fernández and Lamkanfi^1^), they are best known for orchestrating apoptotic cell death during development, normal physiology and disease. Until recently, this ‘executioner’ role of caspases had obscured their non-apoptotic roles, which have now come into focus. Caspases are generated as pro-enzymes and become active after processing. They are best grouped by their function, as either initiator caspases (Dronc, Dredd and Strica in *Drosophila* and caspase-1, -2, -4, -5, -8, -9, -10, -11, -12 in mammals) or effector caspases (Drice, Dcp1, Decay and Damm in *Drosophila* and caspase-3, -6, -7 and -14 in mammals). Initiator caspases have long N-terminal pro-domains and exist as monomers. Upon dimerization, they act on a limited number of substrates, including the effector caspases. In contrast, effector caspases have short N-terminal pro-domains, exist as inactive dimers and have hundreds of targets. Substrate specificity is determined by defined caspase cleavage sites found within target proteins. Depending on the interacting proteins and the initiator caspases involved, there are two main routes to caspase activation: The extrinsic and the intrinsic pathways, both of which converge on the effector, caspase-3.

The key mediators in the extrinsic pathway are death receptors (DRs) that belong to the tumor necrosis factor receptor family. Ligand binding triggers receptor clustering and recruitment of adaptor proteins, which activate pro-caspases. For a review of the extrinsic pathway, see Elmore.^2^

The intrinsic pathway is triggered by mitochondrial outer membrane permeabilization (MOMP), which releases cytochrome *c* (cyt-c). This allows the formation of the apoptosome, an oligomeric platform, containing cyt-c, apoptosis protease-activating factor-1 (Apaf1) and the initiator, Capsase-9. In mammals, MOMP is gated by the pore-forming proapoptotic Bcl2 family of proteins (e.g., Bax, Bak and BH3-only proteins) that are antagonized by the antiapoptotic members of the same family (e.g., Bcl-xL and Bcl2). Bax is predominantly cytoplasmic, translocating to the mitochondria upon apoptotic stimuli. Conformational changes in Bax and Bak allows homo-oligomerization and MOMP. The proapoptotic BH3-only proteins activate Bax/Bak directly or indirectly by inhibition of the antiapoptotic Bcl2 proteins (reviewed in Czabotar *et al.*^3^). Although cyt-c and Apaf1 are present in worms, flies and mammals, their requirement for apoptosome formation and caspase activation varies (reviewed in Kornbluth and White^4^). Activated initiator and effector caspases can be inhibited/ubiquitylated by binding to the inhibitor of apoptosis (IAPs) via their BIR domains.^5^ This IAP-based inhibition is relieved by the IAP-binding motif (IBM) proteins Smac/Diablo and Omi/Htra2 in mammals or by Reaper, Hid and Grim in *Drosophila* that bind to IAP BIR domains via an IBM.^6, 7, 8, 9^

## ‘I’m Not Dead!’ — The Holy Grail

### The non-apoptotic role of caspases

The core apoptotic machinery was largely discovered in *Caenorhabditis elegans*, which dominated the field for a long time. The generation of mutants in *Drosophila* and subsequent *in vivo* studies revealed key non-apoptotic roles for caspases during development. The first non-death role was seen in spermatid individualization that required Dark (dApaf1), Dronc, Dcp1 and Hid.^10, 11^ Around the same time Dronc, but not effector capsases, was found to be required for compensatory cell divisions in *Drosophila* wing discs.^12, 13, 14, 15, 16^ The first non-apoptotic role of caspases in fly nervous system was found in sensory organ precursor development, where Dronc was found to regulate Wingless signalling.^17^ Although caspases had been implicated in tissue remodelling (e.g., megakaryocytes) and axon growth for some time,^18^ the power of *Drosophila* genetics proved to be a ‘watershed’ for elucidating the non-apoptotic roles of caspases. Here we focus on how caspases sculpt postmitotic neurons. (for caspases in cell-fate specification, see review Kuranaga and Miura^19^).

## Caspase Activation During Neuronal Remodelling

Regressive phenomena are crucial for the matching of network components during development and later for circuit refinement. Pruning, the selective elimination of synapses, axons or dendrites, occurs without death of the parent neuron^20^ and its dysregulation has been implicated in disorders, such as schizophrenia^21^ and autism.^22^ It has been defined as either small or large scale, depending on the size of branches removed. Pruning is absent from other organ systems and difficult to recapitulate in a dish, making it one of the least understood cell biological phenomena.

We classify pruning events here as being either macropruning or micropruning. Macropruning refers to removal of intact branches, be they large or small. This occurs either by distal to proximal branch retraction^23^ with axosome shedding^24^ as seen at the vertebrate neuromuscular junction or by a local degeneration, such as in thalamocortical projection neurons where branches are cut and the processes distal to the cut then fragment.^25^ Micropruning events comprise of local structural changes in individual presynaptic boutons or dendritic spines. Most work has focussed on postsynaptic events, where dendritic spines shrink or are removed along a dendritic shaft.^26, 27^

**Figure 1:**
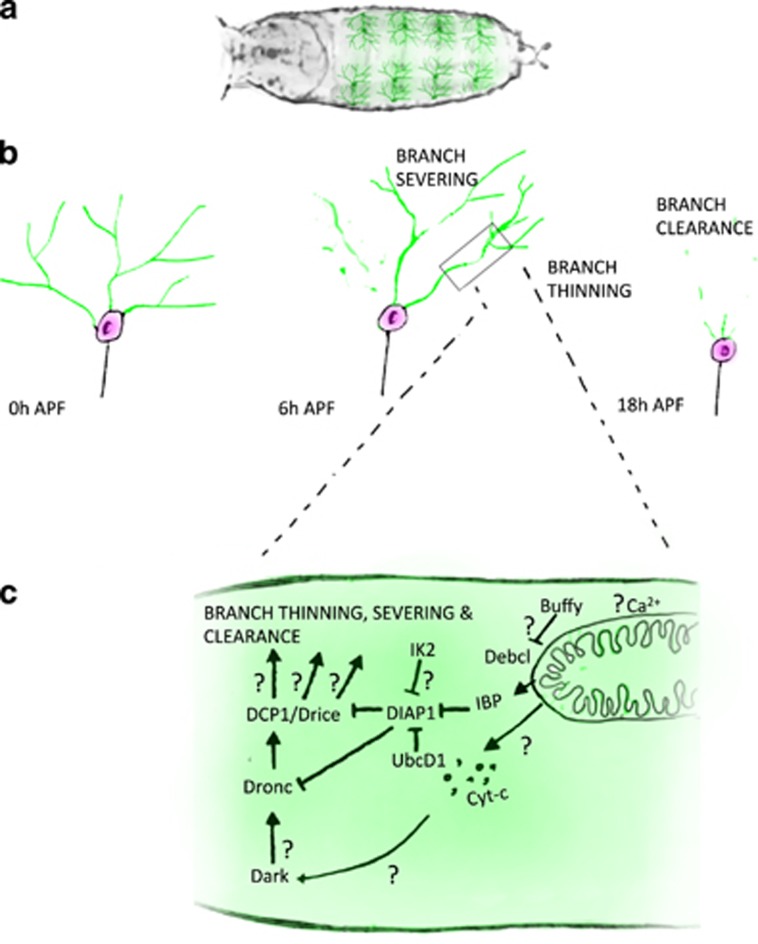
Non-apoptotic role of caspases in dendritic arborization neuron remodelling. (**a**) Overview of Drosophila white pre-pupae (grey) with abdominal dendritic arborization (da) sensory neurons in superficial position on dorsal body wall aspect (green). (**b**) Da sensory neurons undergo remodelling during early metamorphosis. Pruning starts with initial thinning of branches, followed by severing at 6 h after puparium formation (APF), and is completed with the clearance fragments of the severed branches by 18 h APF. (**c**) The apoptotic machinery involved in the pruning of da neurons includes Dark (dApaf1), the fly caspaseDronc, DIAP1 and its regulator UbcD1. The question marks represent the current unknowns in the pathway. Ca^2+^, calcium ions; IBP, IAP-binding motif proteins; IK2, I*κ*B kinase

Insects undergoing complete metamorphosis are excellent models for macropruning as they possess large, identifiable neurons that predictably remodel.^28^ The advent of molecular labelling tools has allowed pruning to be visualized in the nervous systems of small creatures, such as *Drosophila*.^29, 30^ Pioneering studies in *Drosophila* mushroom body *γ*-neurons revealed that axons and dendrites prune by local degeneration^31^ similar to that in vertebrates.^32, 33^ Live-imaging of dendritic arborization (da) sensory neurons, particularly ddaC, the dorsal class IV da, gave unprecedented access to the cellular details of macropruning.^34, 35^ In these sensory neurons, cytoskeletal changes result in thinning of proximal branches, branch severing, fragmentation and clearance of debris by phagocytosis^34, 36^ (Figure 1). When these larval-specific dendrites are removed, adult-specific arborizations begin to regrow in their place.

In the mid-1990s, Raff *et al.*^37^ found no obvious role for caspases in nerve growth factor (NGF)-dependent degeneration of axons. Parallel *in vivo* studies on *pmn* mutant mice overexpressing Bcl-2 suggested that caspases were not involved in dying-back degeneration.^38^ The inhibition of caspases in *Drosophila γ*-neurons also showed no disruption during remodelling.^31^ Nevertheless, live-imaging and the ‘apoptotic-like’ features seen in da sensory neuron pruning^34^ prompted us^39^ and others^40^ to revisit this idea. Using a genetically encoded caspase probe expressed only in pruning neurons, we revealed active caspases within degenerating branches.^39^ In addition, the initiator caspase Dronc, Dark (dApaf1), the DIAP1 (*Drosophila* IAP) along with Effete/UbcD1 and VCP^40, 41^ were found to be required for pruning (see Table 1). The presence of active caspases and a requirement for components of the canonical apoptotic pathway during pruning provided strong evidence for caspases in non-apoptotic roles.

**Figure 2:**
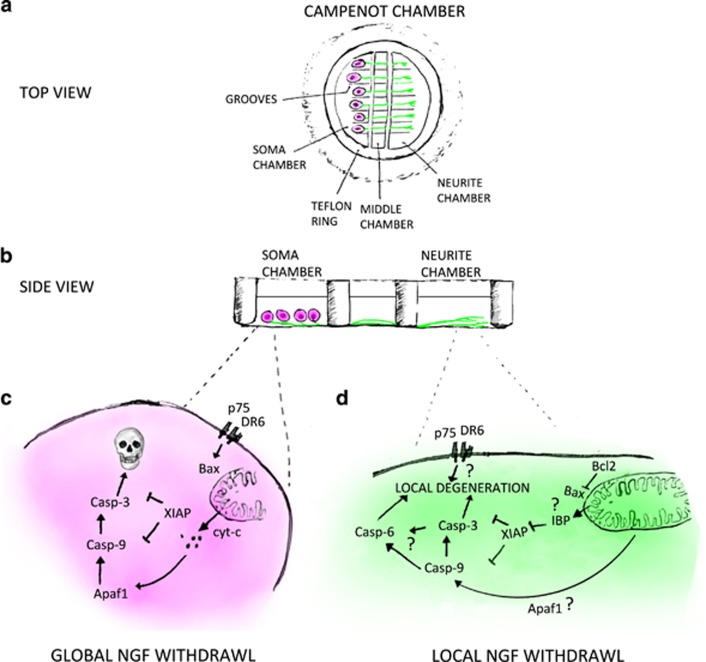
Non-apoptotic role of caspases in NGF-dependent axon degeneration. Axon degeneration and pruning can be studied using a Campenot chamber ((**a**) top view and (**b**) side view). The chamber separates the soma (magenta) and neurite (green) chambers so that cells can be manipulated to either globally or locally deprive neurons of growth factor (NGF). Global withdrawal that results in neuron death (**c**) does not require Caspase-6, which is important for (**d**) local NGF withdrawal-induced axonal pruning. The role of IAP-binding motif proteins (IBPs), Bax, XIAP and Caspases-9, -3 and -6 have all been shown to regulate local NGF-induced axonal pruning. The activation of the pro-caspase 9 without Apaf1 and the mechanism of caspase-6 activation require further investigation. Current unknowns are represented by the question marks. DR, death receptor 6; p75, p75 neurotrophin receptor; IBP, IAP-binding proteins; Casp-9, caspase-9; Casp-3, caspase-3; Apaf-1, apoptotic protease-activating factor 1; XIAP, inhibitor of apoptosis 1 on X; Bax, BCL2-associated X; Bcl2, B-cell lymphoma 2 protein

With this perspective and new genetic tools, the role of caspases in vertebrate axon remodelling was revisited. Using Campenot chambers and microfluidic devices, axon degeneration was reinvestigated with the NGF withdrawal paradigm. These *in vitro* approaches allow either cell bodies or the neurites to be independently exposed to different local environments (Figure 2). The role of various components of the apoptotic machinery were tested using pharmacological and knockdown approaches.^42, 43^ A number of studies have used gene knockouts to confirm that caspase-9 and caspase-3 are essential for axonal pruning and find that caspase-6, BAX and XIAP^44, 45, 46, 47^ are also involved. Many of these are also required for retinal ganglion cell (RGC) pruning, *in vivo*^44^ (see Table 1). An important point to note here is that in full mutants of the apoptotic pathway, the intact axons of ‘undead neurons’ might coexist alongside the ‘unpruned’ axons of remodelling cells, making interpretations difficult. Our work in *Drosophila* has demonstrated that neurons ‘fated to die’ do not always prune their processes by default.^34^ The non-apoptotic role of caspases in macropruning has had many peaks and troughs, but the new data strongly suggest many discoveries lie ahead on the horizon.

## Learning and Memory — Caspases in Synaptic Plasticity

Connectivities established during development are not fixed and can be modified with experience. Such changes, largely taking place at the synapse, are termed synaptic plasticity and are currently considered the strongest candidate mechanism for the biological basis of learning and memory. Long-term potentiation (LTP)^48^ and long-term depression (LTD)^49, 50^ are two of the most well-studied experimental forms of synaptic plasticity in mammals and are believed to model the effects of experience and deprivation on the nervous system. Out of several, the most celebrated form of glutamatergic synaptic plasticity is mediated by two postsynaptic ionotropic glutamate receptors: the NMDA and AMPA receptors.^51^ The NMDA receptor (NMDAR) is a core induction mechanism both for LTP and a form of LTD, serving as a coincidence detector of presynaptic and postsynaptic activity as it requires both ligand and voltage gating. A major expression of NMDAR-dependent LTD is a decrease in synaptic strength resulting from AMPA receptor (AMPAR) internalization at the postsynapse,^52^ with correlated structural change, shrinkage or loss, particularly in juvenile animals.^53^ Application of the agonist NMDA in culture induces a form of ‘chemical LTD’^54^ resulting in AMPAR removal and micropruning, with dendritic spine shrinkage and loss.^27, 55^ These structural changes are correlated with decreased responsiveness to neurotransmitters.

**Figure 3:**
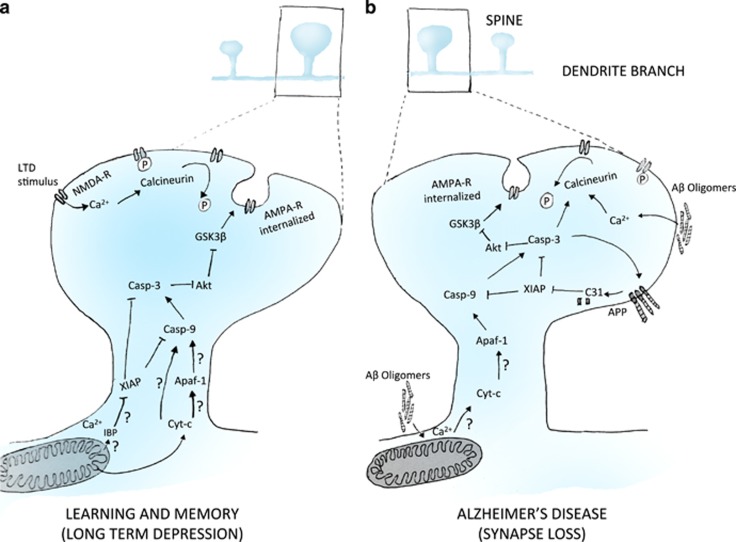
Non-apoptotic roles of caspases in micropruning in plasticity and disease. (**a**) Caspases-9 and -3 along with XIAP, Bad, Bax and antiapoptotic Bcl2 has been shown to be involved in NMDA-R dependent AMPA-R internalization. Active caspase cleaves Akt to release the inhibition of GSK3*β* and LTD. Although cyt-c is released, whether it is required for Apaf1 function and whether the IBM proteins are needed, require clarification. (**b**) In Alzheimer’s disease (AD) presence of A*β* oligomers increases intrasynaptic Ca^2+^ levels to trigger caspase activation. C31 fragment derived from caspase cleavage and enzymatic processing of APP can block XIAP and further contribute to accumulation of active caspases. The requirement of cytochrome c and Apaf1 in the presence of A*β* oligomers is not known. This initial micropruning event can snowball into synaptic loss or dendritic branch loss and contribute to AD. The unknowns are represented by question marks. IBP, IAP binding motif proteins; Ca^2+^, calcium ions; Casp-9, caspase-9; Casp-3, caspase-3; Apaf-1, apoptotic protease activating factor 1; XIAP, inhibitor of apoptosis 1 on X; A*β* oligomers, amyloid beta oligomers; Akt, protein kinase B; GSK-3, glycogen synthase kinase 3; AMPA-R, AMPA receptor; NMDA-R, NMDA receptor; C31, C31 fragment of the amyloid precursor protein; LTD, long-term depression; APP, amyloid precursor protein

Mattson and colleagues first proposed active caspases as a driving force for LTD.^56, 57^ Important support for the role of caspases in learning and memory came from the finding that AMPAR subunits are caspase substrates.^58^ The physiological significance of caspases were first shown when active caspase-3 immunoreactivity was detected in the hippocampus of naive rats.^59^ The active caspase-3 immunoreactivity could also be significantly decreased by injection of caspase inhibitors that resulted in changes to long-term spatial memory storage^59^ and active avoidance learning.^60^ In hippocampal slices incubated with z-DEVD-FMK, a caspase inhibitor, LTP appeared to be suppressed;^61^ however, a later study using slices from caspase-3 knockout mice showed no change in LTP, yet LTD was suppressed.^62^ Elegant *in vivo* studies revealed the spatio-temporal dynamics of caspase-3 activation in the zebra finch during song response habituation.^63^ They found active caspase-3 in the dendritic spines 10–20 min after novel song exposure. Injection of caspase-3 inhibitor prior to song habituation blocked the appearance of persisting memory 24 h after a novel song. In this system, active caspases are bound to XIAP, suggesting that upon stimulation they are released quickly.^63^ Postsynaptic overexpression of Bcl-xL and different fragments of XIAP also inhibited AMPAR internalization and suppressed LTD in rodents.^62^ NMDA treatment of cultured hippocampal neurons induced a rapid and transient increase in active caspase-3, and cyt-c levels peaked within 30 min of NMDA treatment before decreasing to baseline. This brief pulse of active caspase-3 did not result in cell death.^62^ Recently, a study using cultured hippocampal neurons from a XIAP knockout mouse showed reduced synapse number and increased AMPA internalization after NMDA treatment,^64^ providing compelling evidence that XIAP regulates sublethal levels of synaptic caspase-3 activity and has an important role in learning and memory formation.^64^ Support for mitochondrial pathway in NMDAR-dependent LTD was provided using siRNA knockdown and knockout of Bad and Bax in hippocampal slices,^65^ both of which also resulted in a decrease in AMPAR internalization when stimulated with NMDA. In CA1-specific Bax knockout mice, long-term contextual fear memory was impaired although acquisition of fear memory was unaffected.^66^ There is enough evidence to suggest that the role of caspases in micropruning events is critical, but still much about this rapid and fine-scale regulation remains to be understood (Figure 3 and see Table 1).

## Capsase Activation During Progressive Neurological Disease

In the mature and aging nervous system, active caspases are a double-edged blade. The ultimate fate of neurons in progressive neurological disorders is death and caspases are deployed for this function. However, the majority of chronic neurological disorders are fundamentally disorders of the synapses and caspases can often have the not so obvious role of initiators of the degenerative process. Here we focus on the role of caspases in Alzheimer’s and Huntington’s disease (AD and HD, respectively) prior to neuronal cell death.

### Alzheimer’s disease

AD is a neurodegenerative disorder with progressive cognitive decline and memory loss that correlates with dysregulation of early synaptic function and decreased spine density in the AD brain.^67^ The presence of active caspases before evidence of neurofibrillary tangles in tau overexpression mouse models^68, 69^ and increased active caspase-3 corresponding with memory decline in an AD mouse Tg2576 before detection of plaques^70^ suggests non-death-related roles of caspases in AD pathogenesis.

Amyloid precursor protein (APP), one of the key proteins in AD pathogenesis, is alternatively spliced and processed by caspases and other enzymes, releasing four proapoptotic peptides, including A*β* and C31.^71^ Intra-hippocampal inhibition of *γ*-secretase suggested A*β* is important for AMPAR internalization and the structure of dendritic spines.^70^ Interestingly, A*β* also causes preferential endocytosis of synaptic NMDAR,^72^ thus disturbing the synaptic/extrasynaptic NMDAR balance, subsequently reducing pro-survival signalling.^73^ In addition to enhancing LTD, A*β* oligomers also inhibit LTP by activating caspase-3, which cleaves Akt, releasing tonic inhibition of kinase GSK3*β*.^74^ Although excessive A*β* is neurotoxic, at picomolar concentrations it enhances LTP and memory formation,^75, 76^ suggesting a native role. In an Apaf1 null background, Tg2576 mice did not activate caspase-3, suggesting involvement of the mitochondrial pathway.^70^ Other caspases, such as caspase-6 and the ER-resident caspase-4 are also associated with AD,^77, 78^ and activation of the ER stress pathway was observed *in vivo* and *in vitro*.^79, 80^ Proteomics approaches have revealed that caspase-6 substrates include cytoskeletal proteins and others associated with learning and memory,^81^ but a direct evidence of caspase -4 and -6 action in synapses is currently lacking. Caspase-3, -6 or -8 can cleave APP, generating the C31 fragment, whose toxicity depends on its interaction with APP itself.^82^ C31 acts as a potent inducer of apoptosis,^83^ possibly by inhibiting XIAP via an N-term Smac/Diablo-like motif.^84^ Activation of caspases or increased calcium levels upon deposition of A*β*^85^ activates calcineurin-mediated dephosphorylation and internalization of AMPARs. Similar to caspase-3-mediated LTD, activation of GSK3*β* via Akt cleavage may also contribute to AMPAR internalization in AD (Figure 3). GSK3*β* can hyperphosphorylate tau, another known caspase-3 substrate.^86^ Caspase activation by soluble A*β* thus sets off a feedback loop generating more active caspases and toxic protein fragments that can dysregulate and destroy synapses long before neuron death.

### Huntington’s disease

HD is an inherited disease with progressive neuronal damage resulting in cognitive, behavioral and movement dysfunction. It is caused by an abnormal expansion of a trinucleotide repeat coding for Glutamine (Q) in the *huntingtin* (*htt*) gene, and caspase-6 is emerging as the key caspase in HD pathogenesis.^87^ Caspase-3 and -6 can cleave Htt to release the N-terminal poly-Q tract, whose toxicity depends on the length of the poly-Q and its nuclear localization,^88, 89, 90^ which can cause transcriptional upregulation of caspase-1.^91^ Cognitive deficits with defects in synaptic plasticity are an early symptom of HD,^92^ also observed in mouse models.^93, 94, 95^ mHtt caused dendritic spine loss and impaired normal experience-dependent synaptic plasticity in a R6/2 HD mouse model.^96^ WtHtt is known to be anti-apoptotic,^97, 98^ but whether the presence of mHtt directly impacts caspases requires further investigation.

Thus, in addition to acting as substrates for caspases, key proteins in neurodegenerative diseases can also activate caspases causing a ‘snowball’ effect. The recent findings highlight that the non-apoptotic roles that caspases have as initiators of synapse loss, maybe far more important than their role as final mediators of cell death as they provide a target for therapeutic intervention before neuronal loss.

## Regulatory Mechanisms — What’s the Same, What’s Different?

During structural remodelling, neurons need to deploy caspases while maintaining them at sublethal levels. Here we discuss some of the mechanisms that are known to regulate caspases during cell death and speculate on how these may be exploited for non-apoptotic roles.

### Setting the basal levels

Precise *in vivo* manipulations of effector caspase expression in *Drosophila* have shown that the abundance of pro-caspases is directly proportional to caspase activity level,^99^ suggesting the existence of a ‘threshold of activity’ that induces apoptosis. Our recent data show different active effector caspase levels in remodelling neurons *versus* those destined to die during metamorphosis (Mukherjee and Williams, unpublished observations). It is possible that a cell’s identity and age determines its capacity to generate different levels of caspase activity.

One means to regulate the abundance of the core machineries of the apoptotic pathway is via transcription. Pruning in insects is controlled by developmental hormones that signal through the ecdysone receptor EcR/USP,^30^ with downstream targets such as transcription factor Sox-14.^100^ Both Dronc and Drice expression is regulated by steroid hormones via the transcription factor *Broad-Complex* (*BR-C*).^101, 102^ A Dronc promoter fragment showed dynamic levels of expression in the developing nervous system and other tissues.^103^ Interestingly, blocking effector caspases and death in imaginal disc cells maintained high levels of Dronc transcription^104^ suggesting a feedback signal, the mechanism of which remains unknown. Studies on neuronal cell death in vertebrates have mainly focussed on the apoptotic machinery in developing and not mature neurons, which tend to have different response to injury-induced death.^105^ In mature sympathetic neurons, repressive chromatin around the Apaf-1 promoter renders it inactive^106^ and an increased Bcl2 to Bax ratio^107^ likely provides that increased protection from death. It is not known whether caspase activation and regulation in non-apoptotic contexts also differ with a neuron’s age. Recent work in DRG neurons in mammals suggested that transcription of PUMA, a member of the BH3-only subgroup, may activate caspases during axon degeneration.^47^ How this is achieved without the destruction of the soma compartment is an open question.

Studies in *Drosophila* also revealed that the levels of core apoptosome components can be controlled by post-translational inhibitory feedback mechanisms. When the levels of dApaf1 are artificially increased, the levels of Dronc are lowered and *vice versa*, which depends upon Dronc’s caspase recruitment domain and a caspase cleavage site within dApaf1.^108^ Another post-translational mechanism involves de-ubiquitylating enzymes such as DUBA, which stabilizes Dronc, for example, in spermatid individualization in *Drosophila*.^109^ In vertebrates, two different caspase-9 transcripts are generated, one with proapoptotic and the other with antiapoptotic functions. Phosphorylation of caspase-9 also results in reduced activation and/or cleavage of itself.^110^ Although the majority of these mechanisms have been observed in dying cells, they may be deployed to regulate caspase levels in non-apoptotic functions.

### Gating caspases in pruning

**Figure 4:**
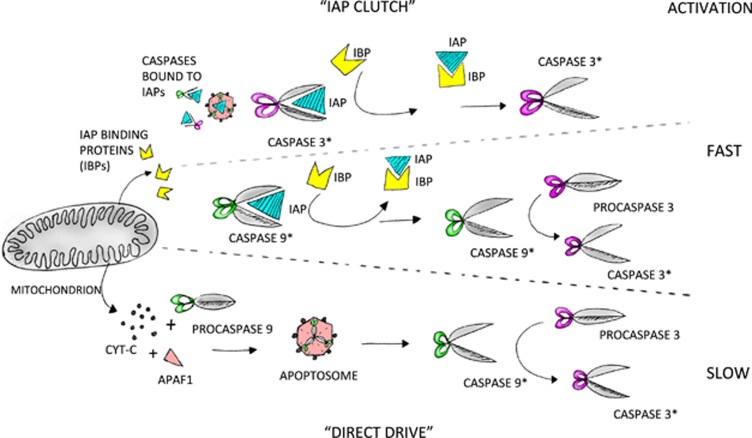
A speculative model of caspase activation in non-apoptotic roles. IAPs could be bound to ProCaspase-9/apotosome complex, activated Caspase-9 and/or activated Caspase-3. The transient and fast activation of caspases in non-apoptotic roles suggests a clutch-like role of IAPs where active caspases are stalled ready to be released and used. In ‘direct drive’ mode, the formation of the apoptosome might overwhelm the IAP clutch and push the system into a slow but more extensive accumulation of active caspases. caspase-3*, activated caspase-3; caspase-9*, activated caspase-9; APAF-1, apoptotic protease-activating factor 1; IAP, inhibitors of apoptosis; IBP, IAP-binding motif proteins

The timescale of macropruning events are similar to cell death, showing peaks in active caspases, lasting anywhere between an hour to a day. In contrast, the morphological changes in cortical neurons following low frequency stimulation reveal that spines can undergo shrinkage/micropruning within 30–60 min following induction.^27^ Similarly, rapid generation of active caspases following novel song stimuli in zebra finches was detected within 10 min.^63^ How are non-apoptotic caspases gated in these two different scenarios? Most of the data suggests involvement of mitochondria, and a number of possibilities exists through which the mitochondrial pathway could be activated. We postulate that one mode is a ‘direct drive’ system, where cyt-c is released with apoptosome formation while the other is through an IAP-based ‘clutch’ system with the active caspases stalled, ready for quick release upon stimuli, bypassing the need for Apaf1 and apoptosome formation (Figure 4).

There seems to be a discrepancy in the requirement of Apaf1 in NGF-dependent axon degeneration,^45, 47^ but whether this arises due to differences in measurement, cell type or mutant backgrounds is unclear. Although more work is needed to address this, it will be useful to establish the requirement of Apaf1 *in vivo* in RGC axon pruning. In flies, dApaf-1 is required for dendrite pruning, yet a direct role of mitochondria and MOMP in *Drosophila* apoptosis is still somewhat controversial. In addition, it is not known whether Apaf1 is required for LTD.

IAPs can bind directly to caspases or ubiquitylate them, either changing their function or targeting them for destruction via the proteasome.^5, 104, 111^ In vertebrates, XIAP is a key IAP that directly binds and inhibits active caspases but cIAP1 can also bind and interact with processed and oligomerized caspase-9 in the apoptosome to block procaspase-3 activation.^112^ Whether cIAP is involved in non-apoptotic caspase functions requires investigation. In *Drosophila* programmed cell death, DIAP1 blocks Dronc activation without targeting it to the proteasome.^104^ Although DIAP1 is important for ddaC neuron pruning,^40^ its exact role remains an open question.

In zebra finch memory formation, active caspase-3 co-localizes and co-precipitates with XIAP. Active caspases can bind reversibly to IAPs.^113, 114^ It is possible that, upon LTD stimulation, a brief loss of XIAP inhibition releases active caspase-3, sufficient to induce LTD and memory but not apoptosis.^63^ The quick and low-level activation of caspases in micropruning suggests that active caspases are likely to be stalled in some complex ready for release upon stimulation. In mammals, XIAP also inhibits the local degeneration of axons following NGF withdrawal. Currently, it is not clear whether such an IAP-based ‘clutch’ system is operating at the level of initiators or effectors or both (Figure 4).

In flies, IAPs are major regulators of the cell death through the complex spatial and temporal expression of the IAP antagonists—the IBM proteins (Reaper, Hid, Grim and Sickle) but it is not known whether they are required during *Drosophila* dendrite pruning. Are the analogous IBM proteins Smac/Diablo and Omi/Htra2 in mammals controlling an IAP-based clutch for non-apoptotic events? Studies in HeLa cells reveal that while cyt-c is released within 5 min, release of Smac take at least 20 min using similar assay.^115^ Although others have suggested a co-release of cyt-c and Smac,^116, 117^ it may differ in different circumstances. For example, in non-apoptotic situations, an ‘incomplete MOMP’ or very low levels of Apaf1 may allow a sublethal caspase activation that does not result in death.

Bax is required in micropruning but its translocation to the mitochondria was not observed, in spite of cyt-c release.^65^ Interestingly, Bax-deficient human prostate cancer cells released Smac and cyt-c in response to apoptotic stimuli,^117^ thus suggesting that Bax may not be essential for MOMP. In non-apoptotic situations that require quick and modest caspase activation, a mechanism of Smac release bypassing Bax translocation could potentially inhibit IAPs briefly and quickly. Although several interesting possibilities exist, the role of mitochondria and the release of IBM proteins need further exploration in different model systems. Currently, it is also unknown whether Smac/Diablo and Htra2 are required for LTD and NGF withdrawal-dependent pruning.

Once caspases are active, IAPs may become crucial to prevent progression into apoptosis. Stabilization of the IAPs may therefore ensure activated caspases are kept at bay. The long form of Fas apoptosis inhibitory molecule (FAIM-L) has an IBM that prevents XIAP auto-ubiquitylation, maintaining its stability.^118^ FAIM-L is almost exclusively expressed in neurons^119^ and has recently been demonstrated to have a role in LTD and in NGF withdrawal-induced axonal degeneration by binding to and stabilizing XIAP.^120^

Overall, the intersection of these data strongly suggest that an IAP-based clutch could be important for controlling activation in a non-apoptotic context in both macropruning and micropruning. Future experiments looking at the dynamics of this *in vivo* will help us gain a clearer picture of the role of IAPs.

### Localization of caspase action

Our previous work has shown that active caspases are restricted to the dendrites of the ddaC neurons^39^ similar to the immuno-EM data of activated caspase-3 in the zebra finch,^63^ suggesting a localized activity. For non-apoptotic roles, if active caspases are to be ‘available on demand’, where are these active caspases maintained ready to be used? Subcellular localization of active caspases in granular structures has been observed in terminally differentiating cells, such as epithelial cells and megakaryocytes.^121, 122^ Whether such granules exist in pruning events is unknown.

In most cells, caspase pro-enzymes are distributed in the cytoplasm while proapoptotic factors are localized in the mitochondria,^123^ which could also be important for keeping the active caspases local. Movement of mitochondria into active synapses during synaptic stimulation has been reported.^124^ The greater spread of LTD from the vicinity of the original stimulus (heterosynaptic LTD^125^) as compared with LTP is also correlated with the presence of mitochondria across multiple postsynaptic sites. If mitochondria are important in restricting sublethal capase action, it would be interesting to follow up the work of Ertürk *et al.* (2014) and move mitochondria into and out of spines, while monitoring the local capsase activation.^126^ The proteasome machinery can also restrict active capsase-3 to the spines.^126^ In *Drosophila,* the UPS and proteasome have been found to be important in mushroom body *γ* neurons^31^ and ddaC sensory neuron pruning.^35^ Alternatively, the local activity of caspases within branches could also be generated by a local inhibition mechanism at the soma, as seen with Tango and Bruce in developing sperm in *Drosophila*.^127, 128^ As yet, it is not known whether these molecules have any role in neuronal pruning.

## ‘The Future ain’t What it Used to be’ — Yogi Berra

The structural remodelling of neurons is a universal phenomenon within nervous systems. A question that emerges from the data is the extent of overlap that exists in the molecular mechanisms that orchestrate macropruning and micropruning events. In the context of caspase activation, the timescales appear to be very different; the rapid release of active caspase-3 in micropruning events suggest it is controlled by an IAP-based ‘clutch’-like mechanism while macropruning can involve transcription and a step-wise activation of caspase-3 via apoptosome formation, that is, more similar to the classical apoptotic cascade (Figure 4).

In order to understand the overlap between caspase-mediated micropruning and macropruning, we should look at the common substrates targeted by effector caspases in both cases. Activated effectors potentially cleave >500 target proteins in humans.^129^ Currently, little is known about caspase targets in non-apoptotic contexts. Structural changes in neurons, both large and small, are the result of cytoskeletal changes, making the cytoskeleton and its regulators some of the most likely candidates. In axon branches, caspase cleaved actin and tubulin fragments are detected locally with epitope-specific antibodies.^130^ However, in case of ddaC sensory neurons, the identity of caspase targets remain unknown.

So far, caspases do not seem to have a role in mushroom body *γ* neuron remodelling. Is this due to the limitation of the tools used or are there parallel caspase-independent programmes of pruning? Surprisingly, active caspases have been found in the axon terminals in the RGCs during their elaboration phase and seem to have a non-destructive role in branch stabilization.^131^ Could these roles be more widespread than we realize? Unlike kinase/phosphatase pathways, protease signalling is an irreversible cleavage-based signal enabling the rapid and simultaneous inhibition or activation of diverse pathways. With multiple nodes at which inputs can exchange information, it should not be surprising that this machinery is used in so many contexts other than death.^132^

Another question that emerges from these observations is whether these non-death roles are ancient or more recent modifications to death programmes? To gain perspective on this, it would be useful to compare molecular mechanisms in a greater number of taxa. Does the emergence of non-apoptotic roles depend on the expansion of regulators and having multiple alternate ways of controlling the pathway? As *Drosophila* and *C*. *elegans* are representatives of the Ecdysozoa, it will be important to gather more information about caspase regulation in the other major Protostome clade, the Lophotrochozoa. Mapping the distribution of caspase targets, within complex tissues/cells that exhibit non-apoptotic processes in different groups, will give insights into evolution of this biology.

One other type of ‘neuritic remodelling’ is Wallerian degeneration. This evolutionarily conserved programme of neurite auto-destruction was revealed upon discovery of the Wlds mouse and the Wlds fusion protein.^133, 134^ Loss-of-function studies in flies and mice revealed native ‘Wlds-sensitive’ machineries.^135^ Although interaction of the Wlds machinery with caspase-dependent pruning has been explored,^136^ more work is needed to understand exactly how these two pathways interface.

Technological innovations will have a major impact on this field in the following years. CRISPR-Cas9 genome editing technology has already facilitated the generation of conditional alleles and multiple gene knockouts and the exploration of caspase biology in non-model organisms is not far away. High-resolution probes and optogenetic techniques will provide insights into the fine spatiotemporal activity of caspases while these new methods for perturbation will help speed discovery.

Thus time is ripe for exploring the cellular, molecular and evolutionary aspects of non-apoptotic caspase function. As we learn more about how these mechanisms construct, modify and ultimately disassemble our nervous system, it is likely that caspases will become as important to the living as they are to the dying and the dead.

## Figures and Tables

**Table 1:**
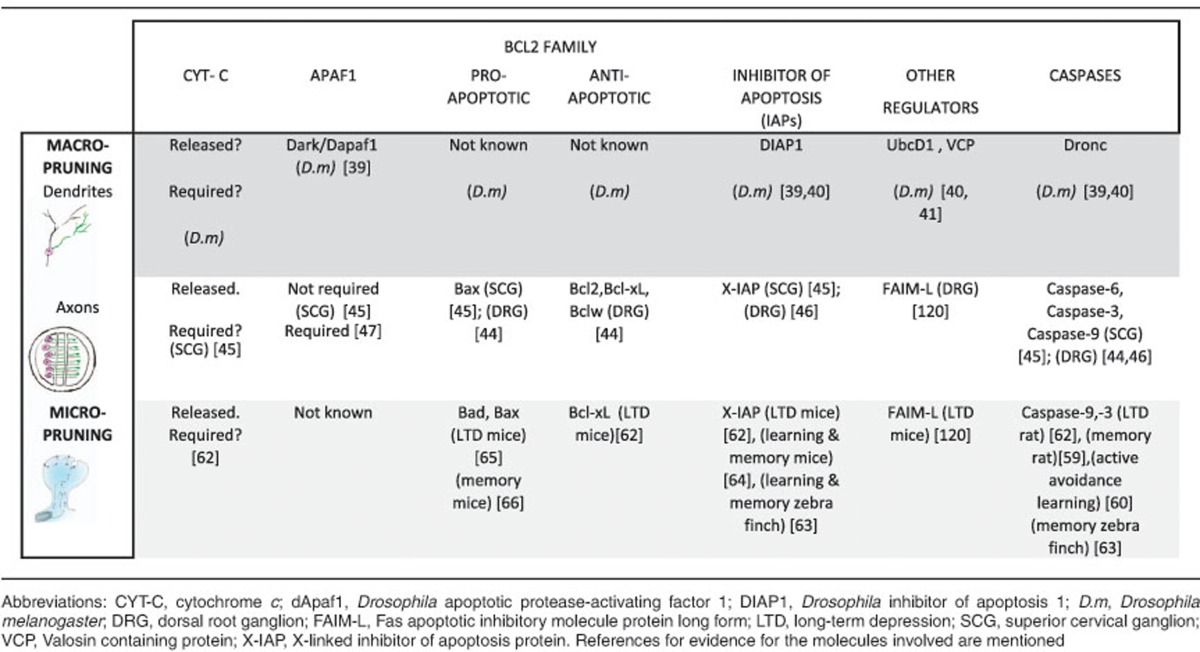
Table summarising the role of caspases and related molecules in dendrite pruning, axon remodelling and learning and memory
